# An unusual Spigelian hernia involving the appendix: a case report

**DOI:** 10.1186/1757-1626-3-22

**Published:** 2010-01-13

**Authors:** Sarah C Thomasset, Eduardo Villatoro, Sarah Wood, Alice Martin, Kelly Finlay, Jane E Patterson

**Affiliations:** 1 Department of General Surgery, King's Mill Hospital, Mansfield Road, Nottinghamshire, NG17 4JL, UK

## Abstract

**Introduction:**

Spigelian herniae are uncommon and frequently pose a diagnostic challenge.

**Case presentation:**

We report the case of a 71-year-old man in whom an ischaemic appendix was found within the sac of a Spigelian hernia during emergency repair.

**Conclusions:**

There are very few reported cases in which an appendix has been found within a Spigelian hernia, in the absence of inflammatory bowel disease. An awareness of the range of viscera which may be encountered in Spigelian herniae is important for safe repair.

## Introduction

Spigelian herniae occur through slit-like defects in the anterior abdominal wall adjacent to the semilunar line. They are uncommon and frequently pose a diagnostic challenge. We report a case in which an appendix was discovered within the sac of a Spigelian hernia during repair. The range of viscera which have been found within Spigelian herniae is discussed. Awareness of possible contents of these herniae is crucial for safe repair.

## Case Presentation

A 71-year old man presented with a 24-hour history of nausea, vomiting and a painful abdominal swelling located in the right lower quadrant. There was no history of a change in bowel habit and he was passing flatus. Past medical history consisted of open repair of an abdominal aortic aneurysm, cerebrovascular disease, diabetes, a myocardial infarct and atrial fibrillation. He was a non-smoker and consumed only a small amount of alcohol.

On examination the patient was comfortable and not tachycardiac or febrile. Abdominal examination revealed the presence of a tender, irreducible mass located between the umbilicus and right anterior superior iliac spine. It was approximately 8 × 8 cm in diameter and had a positive cough impulse. A clinical diagnosis of an incarcerated right Spigelian hernia was made. Abdominal x-rays revealed dilated loops of small bowel.

**Figure 1:**
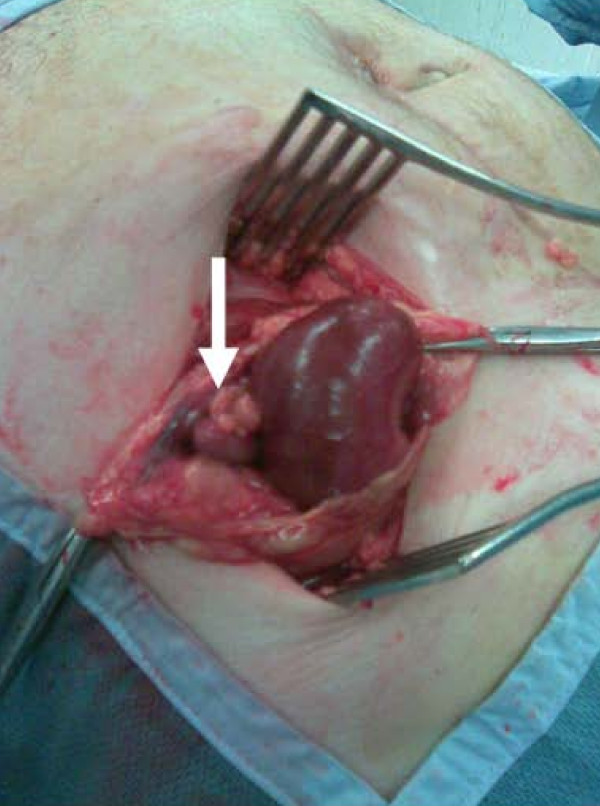
**An ischaemic appendix (white arrow) and loop of terminal ileum within a Spigelian hernia**.

The patient underwent emergency repair of the Spigelian hernia. A transverse skin incision was made over the hernia and the external oblique aponeurosis was divided to reveal the hernial sac which was opened. It contained an ischaemic appendix and a knuckle of small bowel (Figure 1). The appendix was resected and the small bowel, which was viable, was reduced. The small muscular defect was approximated without tension in two layers using polydioxanone (PDS). Postoperative recovery was unremarkable. Histological examination of the appendix revealed features characteristic of ischaemia but no evidence of inflammation or neoplasia.

## Discussion

Spigelian herniae were initially described by Josef Kinkosh in 1764 and named after a Belgian anatomist, Adrian van der Spieghel, who had previously described the semilunar line. They account for 1-2% of all hernias and occur through slit-like defects in the anterior abdominal wall adjacent to the semilunar line. Approximately 90% are located in a 6 cm zone limited superiorly by the transumbilical plane and inferiorly by the interspinal plane. A particularly weak area is the intersection between the semilunar line and the arcuate line of Douglas. The majority of Spigelian herniae are intramural and remain deep to the external oblique aponeurosis. Usual contents are omentum or small bowel, however large bowel, stomach, gallbladder, ovary, testis, bladder, a Meckel's diverticulum and leiomyoma of the uterus although rare, have been described [1]. There are few reported cases in which an appendix has been found within a Spigelian hernia [2,3] some occurring in the presence of Crohn's disease [4,5].

Pain which is exacerbated by contraction of the abdominal musculature is the most common symptom associated with Spigelian herniae and is described by over 60% of patients. The second most common clinical feature is a palpable abdominal mass which is present in approximately 35% of cases [2]. Spigelian herniae characteristically possess a narrow neck (0.5-2 cm in diameter) and at presentation approximately 20% of hernias are incarcerated and 14% are strangulated [6]. Clinical diagnosis is often complicated by the intramural position of Spigelian herniae and the fact that obesity is a predisposing factor. Ultrasonography and computer tomography (CT) may confirm the presence of a hernia in cases of clinical uncertainty.

Treatment of a Spigelian hernia involves primary fascial closure, with synthetic mesh reinforcement if a large defect is identified. Recently, laparoscopic Spigelian hernia repair, using both intra-abdominal and extraperitoneal approaches, has been described. A randomised control trial, albeit involving a small number of patients, has compared outcomes following Spigelian hernia repair. There were no differences in recurrence rates between open and laparoscopic hernia repair, however, laparoscopic repair conferred benefits in terms of hospital stay and morbidity. An extraperitoneal approach was recommended for uncomplicated elective repair and an intra-abdominal approach if co-existent pathology requires surgery during the same intervention. In the case of emergency Spigelian hernia repair an open approach, as performed here, was advocated [7].

## Conclusion

Spigelian herniae are uncommon and frequently pose a diagnostic challenge. A range of viscera may be found within a Spigelian hernia; caution is therefore required when the hernial sac is opened to prevent damage to its contents.

## Consent

Written informed consent was obtained from the patient for publication of this case report and accompanying images. A copy of the written consent is available for review by the journal's Editor-in-Chief.

## Competing interests

The authors declare that they have no competing interests.

## Authors' contributions

All authors approved and equally contributed to this manuscript.
